# Risk Factors Associated with Soil-Transmitted Helminths in Dog Feces That Contaminate Public Areas of Warsaw, Poland

**DOI:** 10.3390/ani14030450

**Published:** 2024-01-30

**Authors:** Agnieszka Tylkowska, Natalia Mocha, Marta Małgorzata Kołnierzak, Magdalena Szenejko

**Affiliations:** 1 Department of Biology of Animal Environment, Institute of Animal Science, Warsaw University of Life Sciences, Ciszewskiego 8, 02-786 Warsaw, Poland; agnieszka_tylkowska@sggw.edu.pl (A.T.);; 2 Department of Environmental Ecology, Institute of Marine and Environmental Sciences, University of Szczecin, Wąska 13, 71-415 Szczecin, Poland; magdalena.szenejko@usz.edu.pl

**Keywords:** dogs, cSTH, helminths, *Toxocara canis*, hookworms, Warsaw

## Abstract

**Simple Summary:**

The increasing number of dogs in towns worldwide may be increasing the risk of environmental contamination by parasites whose growth forms are present in dogs’ feces. Canine, soil-transmitted helminths (cSTHs), most of which have a proven zoonotic potential, are particularly dangerous. In this study, we investigated the presence of cSTH eggs in dogs’ feces left in city parks and dog parks. This study also showed that the presence of dogs’ feces in public areas is still a problem. We observed that dog owners did not pick up their dog’s feces, even though cleaning after one’s dog during walks may be a simple and effective way of limiting the spread of parasitic invasions within the environment.

**Abstract:**

A constant increase in dog numbers, especially in large towns, has been observed recently. The presence of dogs in urban spaces increases the risk of pollution by dogs’ feces, which may contain growth forms of parasites including canine, soil-transmitted helminths (cSTHs), most of which have a proven zoonotic potential. This study assessed the frequency of occurrence and estimated the potential risk associated with the presence of cSTHs in dogs’ feces left uncollected in urban areas. The study material consisted of 200 fecal samples obtained from city and dog parks situated in selected Warsaw districts. Each fecal sample was processed using the flotation technique. Eggs of cSTHs, including *Toxocara canis*, *Toxascaris leonina*, *Trichuris vulpis*, and hookworms from the *Ancylostomatidae* family were found in 23 (11.5%) of the examined fecal samples. The most prevalent species were hookworms from the family *Ancylostomatidae* (8%). The presence of parasites was confirmed in 14 out of 20 studied locations (70%), including eight city parks (72.7%) and six dog parks (66.7%). City and dog parks did not differ significantly in the frequency of parasite occurrence. This study indicated that dogs’ feces, left uncollected, may cause environmental contamination with cSTHs. It also indicated that the presence of dogs’ feces in public areas and the associated presence of parasites is still a problem.

## 1. Introduction

About 85 million domestic dogs are estimated to inhabit Europe [1]. Having an animal gives people the chance to improve their physical and mental health [2,3]. This, however, also increases the risk of animal-borne diseases [4], particularly when many dog owners are not aware of the potential threats [5].

The constantly increasing number of dogs is a serious hygienic, epidemiological, and ecological problem in towns worldwide. Dogs can be a source of pathogens, including parasites [6]. An important factor in the spread of parasites and in the infection of subsequent hosts is the possibility of finding potentially new hosts by means of invasion [7]. Canine, soil-transmitted helminths (cSTHs) are a group of parasites that is present in dogs; they require appropriate external environmental conditions and time to make their growth stages invasive for the next host. Some may cause diseases in people (e.g., *Toxocara canis*, *Ancylostoma caninum*), while others do not harm people but do affect the health of other animals (e.g., *Toxascaris leonina*) [8,9]. The zoonotic potential of *Trichuris vulpis* is still controversial [10].

The growth forms of cSTH spread in the surface soil layer by way of dog feces that contaminate these areas [3,11,12]. Under favorable climatic conditions, the invasive eggs of cSTHs may survive for years, which is particularly dangerous in places frequented by people, like gardens, parks, and playgrounds [8,13]. Infection happens through the accidental consumption of invasive eggs present in the environment [11,14]. Therefore, environmental contamination by dog feces containing the growth stages of parasites is the main source of infection in people and animals [15].

In towns, dogs defecate in green areas that are also visited by people. Green areas may include various recreational, social, and sport locations used by both children and adults [16,17]. If dog owners do not remove their pets’ feces, these areas may become a source of contagious factors, including cSTHs that infect other dogs, wild animals, and people [18].

Prophylaxis for pets may improve the health status of dogs and restrict the spread of animal-borne diseases among people [19,20]. The occurrence of parasites in dogs depends on many factors, including the natural resistance of a given individual, the natural habitat of the animals, and the actions undertaken by owners. The last exerts an important effect on limiting parasites in dogs. Therefore, one of the significant aspects of prophylaxis is educating animal owners [21]. There are, however, factors restricting prophylactic actions by dog owners: for example, poor education, limited financial resources, living in poor areas, and limited access to veterinary services [22,23,24].

One of the important ways for preventing the occurrence of parasites in dogs is the removal of pets’ feces. The proper removal by owners of dog excrement in public places may decrease the environmental contamination caused by the eggs of parasites [25]. In this way, infections can be prevented in people, particularly in children, whose normal behavior (such as touching their face with their hands) make them most vulnerable to infection [7,26]. Unfortunately, despite a range of educational activities, uncollected dog feces are still a serious problem in urban areas.

An additional problem is created by dogs not being under human control. A lack of veterinary supervision and free roaming result in a significant potential for the transmission of parasitic diseases [27]. Studies on the presence of parasites in dogs usually pertain to domestic animals, but these studies do not consider the degree of infection in stray dogs [28].

The application of simple and effective prophylactic actions is important in preventing parasitic invasions. Such actions may include the use of gloves when removing dog feces, preventing animals from accessing sandpits, and observing basic hygienic rules (such as washing hands and food) [29].

Veterinarians can play a significant role in restricting the occurrence of parasites in dogs. By educating their clients about the risks associated with parasitic diseases and about effective methods for their prevention, they can contribute to increasing social awareness of the need for cleaning up dogs’ excrement, having regular parasitological tests, and de-worming pets if necessary [30,31]. A statistically significant relationship was found between dog owners who visited veterinary clinics once or twice a year and who properly removed their dog’s feces, and those owners who used veterinary services only in an emergency [32].

The main method employed for the treatment and prevention of parasites in dogs is the application of de-worming remedies [33]. The risk of infection by parasites increases in dogs that are not regularly de-wormed [34,35,36]. It is, however, the animal owners’ responsibility as to whether their pets are regularly checked and treated. Despite various de-worming agents being readily available, their application is limited due to a lack of education about the use of the drugs and the risk factors associated with the transmission of parasites [37]. In a questionnaire survey carried out among dog owners in Ireland, 52% of respondents declared that they de-wormed their animals once or twice a year or not at all. Moreover, 13% of respondents living in towns admitted that they had never treated their dogs [31]. The so-called prophylactic use of de-worming agents is also common practice. This approach increases the risk of parasites developing a resistance to drugs [38]. The prophylactic de-worming of dogs, even three to four times a year, does not guarantee total protection from parasites [39,40]; therefore, an individual approach to each case, including taking into consideration the risk and physiological status of the dog, is important [38]. Educating dog owners about the importance of de-worming may positively affect owners’ behavior and decrease the risk of infection [25,32,35]. A statistically significant, positive relationship was found between the frequency of visits to veterinary clinics and the dog owners’ adherence to the rules for de-worming dogs. This result suggests that veterinarians may affect the dog owners’ awareness of the risks of parasite infection [32].

The aim of this study was to estimate the frequency of occurrence and determine the risk associated with the presence of cSTHs in dog feces in city parks and dog parks in the northwestern districts of Warsaw.

## 2. Materials and Methods

### 2.1. Study Area

The study material was collected from public green areas. The 20 selected areas included 11 city parks and nine dog parks (green fenced areas where dogs can roam without a lead or muzzle, under the supervision of their owners) (Table 1). The study areas were localized within the administrative boundaries of four northwestern districts of Warsaw: Bielany, Bemowo, Żoliborz, and Wola.

The surface area of the district, the percentage share of green areas based on data from the Statistics Poland 2021 (https://bdl.stat.gov.pl/bdl/start, accessed on 15 October 2021), and the availability of city parks and dog parks were considered when selecting the number of green areas in each district. According to these data, Bielany had the largest surface area (32.34 km^2^, with 11.69% occupied by green areas, including 7 city parks), followed by Bemowo (24.95 km^2^, with 10.37% occupied by green areas, including 4 city parks), then Wola (19.26 km^2^, with 17.2% occupied by green areas, including 5 city parks), and finally Żoliborz (8.47 km^2^, with 29.69% occupied by green areas, including 6 city parks).

### 2.2. Sample Collection and Coprological Analysis

From July to October 2022, 200 fresh canine fecal samples were collected—10 samples from each park. The freshly collected samples were packed in plastic bags with labels stating the location, the number of the sample, and the date. The samples were placed in a refrigerator and later examined in the laboratory. Each sample was processed using a flotation technique (saturated NaCl solution, 1.20 specific gravity) [41,42]. Each sample was microscopically examined at 100× and 400× magnifications. Egg identification was performed using morphological references [43]. Samples were classified as positive if the presence of eggs was confirmed [44].

### 2.3. Data Analysis

Basic parasitological parameters were calculated and defined according to Bush [45]. The prevalence was estimated using the percentage of positive samples among all tested samples of dog feces. The software package Statistica v. 13.1 for Windows was used for the statistical analysis of the data (StatSoft, Inc., Tulsa, OK, USA, 2013). Attributes were represented via frequencies and percentages. Chi-squared (χ^2^) frequency and Fisher’s tests were applied to analyze the differences between attributes. The relationships between the prevalence of the examined dog feces samples, the nature and purpose of the analyzed parks (city parks, dog parks), and their location in various districts of Warsaw (Żoliborz, Bielany, Wola and Bemowo) were examined. A *p*-value of less than 0.05 (*p* < 0.05) was considered statistically significant.

## 3. Results

Microscopic analyses revealed the presence of eggs of *Toxocara* spp., *Toxascaris leonina*, *Trichuris vulpis*, and hookworms of the family *Ancylostomatidae* (Table 2).

The most prevalent species were hookworms from the family *Ancylostomatidae* (8%), followed by *Trichuris vulpis* (3%) (Table 2).

From among the 200 analyzed fecal samples, 23 (11.5%) contained the eggs of parasites. Eleven of these samples (10%) were collected from city parks and 12 (13.4%) from dog parks. No statistically significant difference was found between the frequency of the occurrence of dog parasites (infected and healthy dogs) and the types of parks analyzed (χ^2^ = 0.44, df = 1, *p* = 0.658) (Table 2); hence, no difference in parasite prevalence between city parks and dog parks was confirmed.

Hookworms from the family *Ancylostomatidae* had the greatest share among the noted cSTH species, for both city parks and dog parks (5.45% and 11.1%, respectively; Table 2).

The presence of parasites was noted in 14 out of the 20 studied locations (70%), including eight out of 11 (72.7%) city parks and six out of nine (66.7%) dog parks. No significant differences were noted in the presence of parasites in the dogs’ feces between the particular study districts in Warsaw (χ^2^ = 7.57, df = 3, *p* = 0.056) (Table 3). Three samples showed one type of co-infection (Table 3).

Wola was the district where the highest percentage of infected dogs was found. In both city parks (χ^2^ = 10.69, df = 3, *p* = 0.014) and dog parks (χ^2^ = 66.67, df = 3, *p* = 0.000), the greatest prevalence values (20% and 40.0%, respectively) were noted in Wola (Table 3). Moreover, in the park areas of the district, the frequency of representatives of *Ancylostomatidae* gen. sp. was significantly higher compared to other parasites (Table 4).

**Figure 1:**
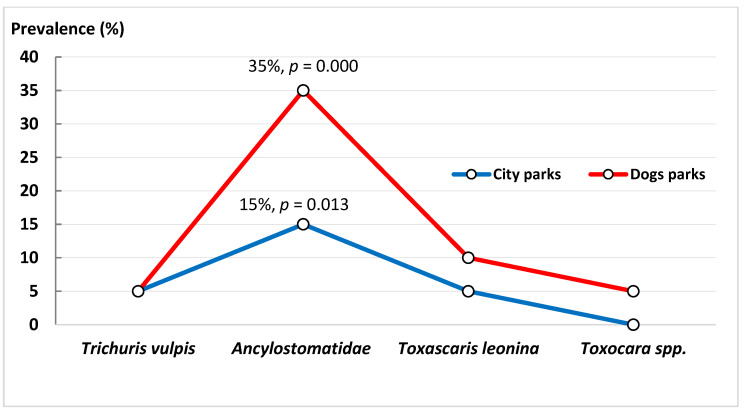
The relationship between the percentage of infected hosts and the type of park in the Wola district. A significantly higher prevalence of representatives of *Ancylostomatidae* gen. sp. was detected compared to other identified parasites.

The prevalence of *Ancylostomatidae* in the analyzed city parks of the district of Wola was about 15.0% (χ^2^ = 8.73, df = 2, *p* = 0.013), whereas in the dog parks, the prevalence was 35.0% (χ^2^ = 37.20, df = 2, *p* = 0.000) (Figure 1). The difference in prevalence between the two types of sites was statistically significant (χ^2^ = 10.67, df = 1, *p* = 0.002). Therefore, a higher percentage of hosts infected by *Ancylostomatidae* was noted for dog parks in the district of Wola. Of note, *Toxocara* spp. was excluded from statistical analysis since it was found in only one dog park and in one host, and was not found at all in city parks.

## 4. Discussion

The overall apparent prevalence of cSTH eggs that contaminated fecal samples in Warsaw was 11.5%. In Poland, similar results were obtained in earlier studies in Warsaw (18.8%) [46] and Olsztyn (19%) [47]. However, again in Poland, a higher prevalence of parasites was found in Łódź (29.5%) [48] and in Szczecin (34.8%) [49]. According to information from the available literature, different degrees of contamination by helminth eggs and larvae determined from canine feces have been established for Europe—from 8.6% in Italy [50] to 71.4% in Spain [51] and 75.7% in Albania [52]. The problem in comparing such results is that studies in a given area are performed rarely and comparisons between far-distant populations may be not reliable.

The differences in research results across the world may be attributed to various factors, such as different diagnostic techniques [53] or the socioeconomic status of the countries where the research was conducted (veterinary control and animal care, hygienic standards) [13,54]. Taking into account that the samples analyzed here were from the environment and that the status of each definitive host was unknown, associating results with the dogs’ characteristics (e.g., age, gender, breed, and underlying living conditions) and with the owners’ conduct of care, i.e., veterinary care and antiparasitic treatment, may not be possible [55,56]. Moreover, there is a chance that the analyzed feces samples were from other, similar-sized animals like foxes, whose presence has been noted in Warsaw.

Numerous studies on dog feces describe discrepancies in the diagnostic efficacy of different parasitological techniques [57,58]. According to Stefański [43], none of the techniques described in the literature can ensure the detection of all types of parasites. The procedure used in this study for detection of cSTH eggs was the common fecal flotation method in a saturated sodium chloride solution (NaCl) with a specific gravity of 1.20 g/mL [41,42]. This method is simple, relatively inexpensive, and effective, particularly for recovering light eggs (e.g., hookworms from the family *Ancylostomatidae*) [43]. However, it also has some limitations that influence its accuracy. Testing for the presence of heavy (e.g., ascarids: *Toxocara* spp. and *Toxascaris leonina*; trichurids: *Trichuris* spp.) or operculate (e.g., flukes) eggs often produces false negative results, because heavy eggs do not float well [43]. Moreover, saturated NaCl solution causes the lysis of most intestinal protozoa cysts (e.g., *Giardia* sp.) [59]. Therefore, it is necessary to take into account that in this study, the prevalence of cSTHs may have been underestimated.

The occurrence of cSTH eggs (*Toxocara* spp., *Toxascaris leonina*, *Trichuris vulpis*, and hookworms of the family *Ancylostomatidae*) was noted in this study. If we look at studies conducted worldwide, we can see that these taxa are globally most frequent in dogs and that they are the most common contaminants of the urban areas [11,13,14,35,55,56,60,61,62,63]. Apart from the effect on the dogs’ health, most cSTHs also have a zoonotic potential [64].

Analyzed samples most often contained the eggs of hookworms of the family *Ancylostomatidae* (8.5%). The prevalence of hookworms of the family *Ancylostomatidae* in dogs in Poland has been reported as being 3.0–7.4% in central Poland [48,65], 13.1–16.2% in northern and northwestern Poland [49,66], and 22.2% in central and southern Poland [67]. Globally, hookworms are the most-often diagnosed internal parasites in dogs [68]. Some studies have found an increased risk of hookworm infection in dogs when dog feces have not been disposed of in a timely or proper way, thus contributing to the development of infective hookworm larvae and environmental contamination [34,35,69].

While hookworms were frequently reported in this study, the zoonotic roundworm *Toxocara* spp. was detected with a prevalence of 0.5%. Eggs of *Toxocara* spp. were found in one fecal sample from a dog park in the district of Wola. A similar result was obtained in studies carried out in Australia, where *T. canis*. was detected with a prevalence of less than 0.5% in dogs [35]. Although its prevalence appears negligible, a significantly higher rate of egg laying occurs in puppies and dogs less than 1 year old, while confirmed infections are less common in adult dogs owing to the somatic migration of larvae [70]. Despite the relatively low prevalence in canine fecal samples, seroprevalence of toxocariasis in humans in Australia has been estimated at 7%, indicating that people’s exposure to the highly resistant infectious stage of *Toxocara* spp. is high [71]. Moreover, in the current study, due to the low number of city parks and dog parks studied (N = 20), one cannot estimate the degree of contamination by the eggs of *Toxocara* spp. in the public areas in Warsaw. Further studies on a greater number of locations across all districts of the city are needed. In previous studies conducted in Poland, higher results have been obtained: 4.1–16.8% in central Poland [48,65], 6.5–23.4% in northern and northwestern Poland [49,66], and 7.2% in central and southern Poland [67].

*T. canis* is a common roundworm parasite found in dogs and has a worldwide distribution [72]. There are many studies on the prevalence of *Toxocara* infection in dogs globally. The prevalence of *T. canis* in dogs has been reported as being 1.2% in Australia [35], 4.4% in the Netherlands [73], 4.6% in Belgium [74], and 6.1% in Germany [56]. In some surveys, in countries such as Portugal, Nigeria, India, and China, the prevalence was found to be as high as 51–100% in puppies and 1–45% in adult dogs [72,75,76,77].

Geographic location, outdoor access, and behavior while outdoors are also key factors influencing parasitic infection of domestic pets. The risk of transmission of *Toxocara* eggs was found to increase in free-roaming, unleashed dogs [78]. Dogs that roamed regularly transferred the eggs of *T. canis* on their paws, while their owners did the same on shoes [79]. Coprophagy and rolling around in the feces of other animals may also increase the risk of infection by *T. canis* [80]. Hence, the access of *T. canis*-infected dogs to parks, playgrounds, and recreational spaces has been identified as the main factor influencing the global prevalence of human toxocariasis [81]. However, the influence of this factor could drop significantly upon compliance with a de-worming regimen and cleaning up dog feces [82].

If ingested, the eggs of *T. canis* can pose a health risk to humans, as well as being of veterinary significance [83]. Toxocariasis that is caused by *T. canis* is the most common animal-borne parasitic disease found in humans [84]. Recent epidemiological research has estimated that approximately 1.4 billion people worldwide, particularly in subtropical and tropical regions, are infected with, or have been exposed to, *Toxocara* sp. [85]. The reported seroprevalence of the infection in different geographic regions varies from 2.4 to 92.8% worldwide [86,87]. Some human risk factors, especially in children, have highlighted the importance of soil and it being the main source of infection in the spread of toxocariasis [88]. Environmental contamination with *Toxocara* eggs is common in public places in most countries [89,90,91].

The presence of *Toxascaris leonina* (1.5%) was also noted in this study. In Poland, similar results were obtained in earlier studies for Warsaw (0.7%) [65], Łódź (1.1%) [48], and Szczecin (2.3%) [49]. Generally, *T. leonina* displays lower prevalence in dogs than *T. canis* [92]. However, in this study, *T. leonina* was found more often than *T. canis*, although both species were characterized by low prevalence. This very low prevalence of *T. leonina* is consistent with what has been estimated for dogs worldwide (2.9%), and can be attributed to the limited routes for *T. leonina* transmission among dogs compared to *T. canis* [92]. *T. leonina* is one of the cSTH species devoid of zoonotic potential. The accidental infection of people is, however, possible—several cases of toxocariasis have been reported globally [93,94].

*Trichuris vulpis* is common in Europe, being one of the most frequent alimentary tract parasites in dogs. Depending on the country and the population being studied, from 0.2% to as many as 60% of dogs are infected [95]. *T. vulpis* eggs survive for long periods in the environment, especially in temperate climates [10], where they become a constant source of infection and often lead to high infection rates in dogs. Reports from Belgium and Holland have found that *T. vulpis* is the second most common helminth [96,97]. In our study the prevalence of *T. vulpis* in dogs was 3%, which was similar to the prevalence found by earlier studies conducted in Warsaw (3.1%) [65]. A survey in Spain found that 1.66% of dogs were infected with *T. vulpis* [51]; other studies found infection rates of 10–18% in Italy [98], 10.93% in Serbia [61], and 13–48% in Hungary [99]. Older dogs tend to be more often infected with *T. vulpis* [100]. *T. vulpis* has previously been reported as a cause of visceral larva migrans (VLM) and as an intestinal parasite in humans. However, it is not commonly considered a zoonotic nematode in pets, despite a few reported cases of human infection [10,100,101].

In the current study, the presence of cSTH eggs was noted in 14 out of the 20 studied locations (70%), including eight out of 11 city parks (72.7%) and six out of nine dog parks (66.7%). Similar results were obtained in studies performed in other European countries and in Australia, where cSTHs were found in almost half of the urban green areas being studied [16,17,62]. In the current study, no statistically significant differences were found in the frequency of parasites between city parks and dog parks. However, due to the dog parks having a much smaller area compared with that of the city parks, the dogs’ risk of infection with parasites may be higher in the former.

## 5. Conclusions

The presented study indicates that dog feces in city parks and dog parks in selected districts of northwestern Warsaw may be the reason for environmental contamination with cSTHs, most of which have a proven zoonotic potential. Performed studies showed that the presence of dogs’ feces in public areas and the associated presence of parasites are still a problem. Dog owners were observed not cleaning up the excrement of their animals, even though cleaning during dog walks is a simple and effective way to restrict the spread of parasitic invasions in the environment. Therefore, educational activities are necessary to increase the awareness of dog owners regarding the risks associated with the presence of parasites in public areas.

It is particularly important to carry out further studies to monitor the presence of dog parasites in public areas, especially in densely populated areas and in those visited by children. The results of such studies should be presented to local authorities. This might persuade local authorities to undertake actions aimed at protecting the environment from contamination and, consequently, limiting the risk of parasitic invasions.

## Figures and Tables

**Table 1:** The number of selected city parks and dog parks in particular districts of Warsaw.

City District	The Number of City Parks	The Number of Dog Parks	Total
Bielany	5	4	9
Bemowo	1	2	3
Wola	2	2	4
Żoliborz	3	1	4
Total	11	9	20

**Table 2:** The prevalence of canine soil-transmitted helminths (cSTHs) found in dog fecal samples.

cSTH	Number of Positive Samples (Prevalence, %)		
	City Parks (N = 110)	Dog Parks (N = 90)	Total (N = 200)
*Toxocara* spp.	0	1 (1.1)	1 (0.5)
*Toxascaris leonina*	1 (0.9)	2 (2.3)	3 (1.5)
*Trichuris vulpis*	4 (3.63)	2 (2.3)	6 (3)
Hookworms from the family *Ancylostomatidae*	6 (5.45)	10 (11.1)	16 (8)
Total	11 (10)	12 (13.4)	23 (11.5)

**Table 3:** Prevalence of canine, soil-transmitted helminths (cSTHs) found in dog fecal samples collected in the examined districts of Warsaw.

City District	City Parks		Dog Parks	
	n/N	P	n/N	P
Bielany	4/50	8	2/40	5
Bemowo	1/10	10	1/20	5
Wola	4/20 *^	20	8/20 **^^	40
Żoliborz	2/30	6.7	1/10	10
Chi-squared test	χ^2^ = 10.69 *p* = 0.014		χ^2^ = 66.67 *p* = 0.000	

n—number of positive samples; N—number of examined samples; P—prevalence (%). *—statistically significant at *p* < 0.05; **—statistically significant at *p* < 0.01. ^ There was one sample that presented co-infection: two between hookworms from the family *Ancylostomatidae* and *T. vulpis*. ^^ There were two samples that presented a co-infection: two with hookworms from the family *Ancylostomatidae* and *T. leonine*, and three with hookworms from the family *Ancylostomatidae*, *Toxocara* spp. and *T. leonina*.

**Table 4:** Evaluation of the prevalence and the number of analyzed dog feces samples (n) in city parks and dog parks in the district of Wola.

cSTH	The Number of Positive Samples (Prevalence, %)	
	City Parks (N = 110)	Dog Parks (N = 90)
*Toxocara* spp.	0	1 (5)
*Toxascaris leonina*	1 (5)	2 (10)
*Trichuris vulpis*	1 (5)	1 (5)
Hookworms from the family *Ancylostomatidae*	3 (15 *)	7 (35 **)
Total	4 (20)	8 (40)

N—the number of examined samples. *—statistically significant at *p* < 0.05, **—statistically significant at *p* < 0.01.

## Data Availability

All data generated or analyzed during the study are included in this published article. The datasets used and/or analyzed in the current study are available from the corresponding author upon request.
